# Construction and effect evaluation of different sciatic nerve injury models in rats

**DOI:** 10.1515/tnsci-2022-0214

**Published:** 2022-03-07

**Authors:** Qu Siwei, Ning Ma, Weixin Wang, Sen Chen, Qi Wu, Yangqun Li, Zhe Yang

**Affiliations:** 2nd Department, Plastic Surgery Hospital, Chinese Academy of Medical Sciences and Peking Union Medical College, No. 33 Badachu Road, Shijingshan District, Beijing 100144, China

**Keywords:** sciatic nerve injury model, transverse injury, chemical injury

## Abstract

**Background:**

The most commonly used experimental model for preclinical studies on peripheral nerve regeneration is the sciatic nerve injury model. However, no experimental study has been conducted to evaluate acute injury modes at the same time.

**Objective:**

We conducted sciatic nerve transverse injury, clamp injury, keep epineurium and axon cutting injury, and chemical damage injury in rats to evaluate the degree of damage of the four different injury modes and the degree of self-repair after injury.

**Methods:**

The sciatic nerve transverse injury model, clamp injury model, keep epineurium injury model, and chemical damage injury model were constructed. Then, the sciatic nerve function was assessed using clinical evaluation methods and electrophysiological examinations, as well as immunofluorescence and axonal counting assessments of the reconstructed nerve pathways.

**Results:**

The evaluations showed that the transverse group had the lowest muscle action potential, sciatic functional index, nociceptive threshold, mechanical threshold, rate of wet gastrocnemius muscle weight, area of muscle fiber, and numbers of myelinated nerve fibers. The chemical group had the highest, while the clamp group and the keep epineurium group had medium.

**Conclusion:**

Transverse injury models have the most stable effect among all damage models; chemical injury models self-recover quickly and damage incompletely with poor stability of effect; and clamp injury models and keep epineurium injury models have no significant differences in many ways with medium stability.

## 1 Introduction

Peripheral nerves are nerve tissues which are present outside the brain and the spinal cord. Peripheral nerve injury is a common and potentially harmful clinical injury, with an average of 13–23 cases per 100,000 people per year [1]. Traumatic causes of peripheral nerve injury include penetrating injury, traction or compression, ischemia, shock, and vibration injury. Traction-related injuries, sharp object lacerations, or long bone fractures caused by motor vehicle accidents are the most common causes of injury in life [2]. Many patients treated for peripheral nerve injury still show incomplete recovery during long-term follow-up, often accompanied by partial or total loss of motor, sensory, and autonomic nerve function, as well as intractable neuropathic pain [3]. In general, peripheral nerve injury not only brings great physical defects and psychological pressure to patients but also causes a serious economic burden to society [4,5]. Therefore, it is meaningful to focus on exploring the mechanism of nerve tissue regeneration and developing effective treatments for peripheral nerve injury.

Due to the complex structure of the nervous system, it is difficult to replicate *in vitro*, so research on peripheral nerve regeneration has been very limited and carried out in animal models [6]. The most commonly used experimental model for preclinical studies on peripheral nerve regeneration is the sciatic nerve injury (SNI) model [7,8,9]. The SNI model has the following advantages: the sciatic nerve is large and convenient for surgical operation; the sciatic nerve anatomy is clear, easy to construct surgical channels, and provides a good surgical field. As the SNI model is used in a large number of studies, data can be summarized from previous experiments to optimize our experimental research, and more accurate conclusions can be drawn by comparison [10]. There are many methods for SNI, which can be simply classified into physical injury and chemical injury. Physical damage is most frequently used, and transverse damage [11] and clamp damage [12] are most commonly used in physical damage, in addition to freezing damage [13], compression damage [14], and traction damage [15]. However, no experimental study has been conducted to evaluate acute injury modes at the same time. Because freezing injury is seldom used, compression injury is a chronic injury, and traction injury is not easy to quantify, so these three methods were not selected in our experiment. We conducted sciatic nerve transverse injury, clamp injury, keep epineurium and axon cut injury, and chemical damage injury in rats at the same time to evaluate the degree of damage of the four different injury modes and the degree of self-repair after injury.

## 2 Materials and methods

### 2.1 Animals

Fifty male Sprague Dawley rats (3 months old, weighing 180–200 g) provided by Beijing Vital River Laboratory Animal Technology Co., Ltd were used in this study. The rats were housed in specific pathogen-free cages under an artificial cycle of 12 h of light and 12 h of darkness.

Rats were randomly divided into the following five groups (*n* = 10 each): injury without sciatic nerve (control group); sciatic nerve was transected (transverse group); sciatic nerve was clamped by a microneedle holder (clamp group); sciatic nerve epineurium was retained and the tissue within it was transected (keep epineurium group); and sciatic nerve was injected with lysophosphatidylcholine (LPC) (chemical damage group).


**Ethical approval:** The research related to animals’ use has been complied with all the relevant national regulations and institutional policies for the care and use of animals. This animal study was reviewed and approved by the Local Animal Ethics Committee (202003003).

### 2.2 Surgical procedure

Surgeries were performed by an experienced surgeon under a neurosurgical microscope (M400-E, Leica, Germany). Ten rats across the five groups (two rats per group) underwent surgery each day. The rats were randomly selected from each group, and the order of the surgeries was randomized.

**Figure 1:**
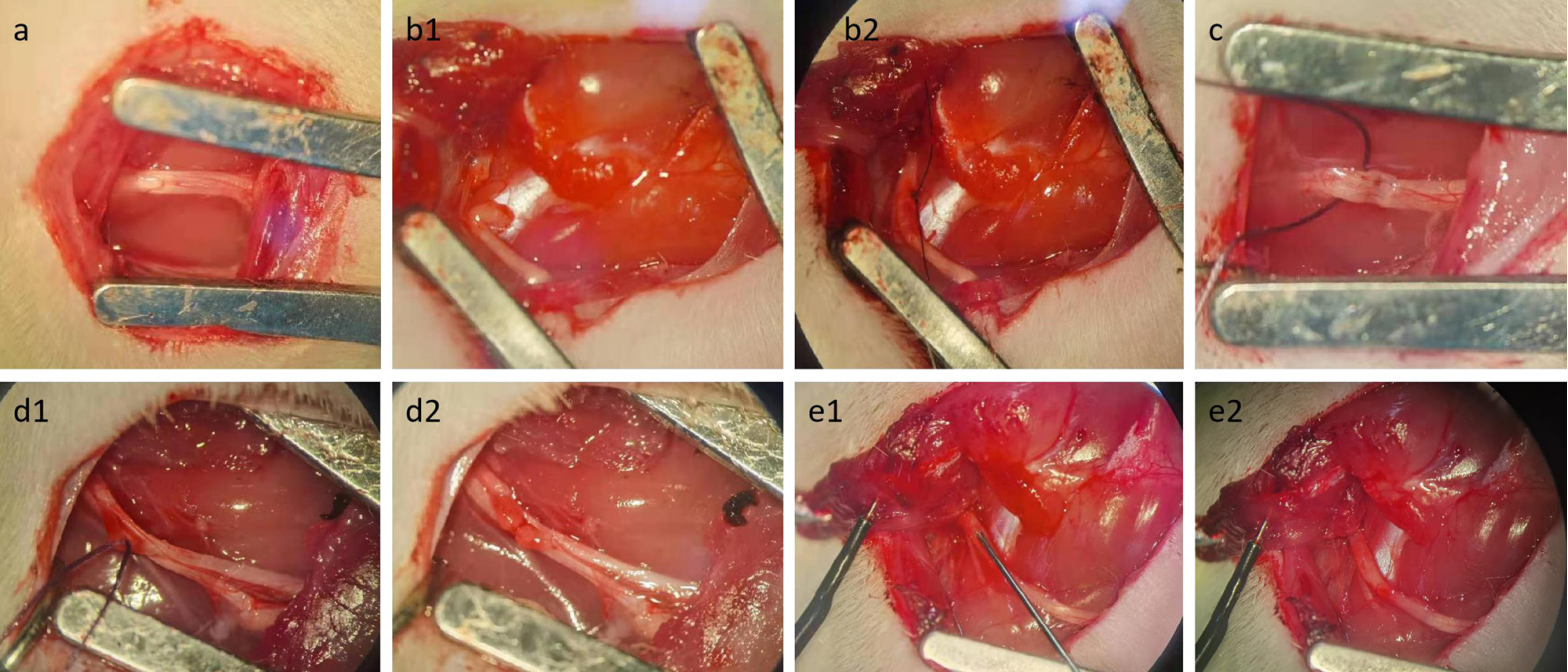
Model construction of different injury modes of the sciatic nerve. (a) The sciatic nerve in the control group was not subjected to any operation. (b1) The sciatic nerve was cut. (b2) The two broken ends are brought together and fixed with sutures. (c) A crush lesion was inflicted at the sciatic nerve trunk, and this procedure left a 1 mm-wide gap. (d1) The sciatic nerve epineurium was slit longitudinally. (d2) Axons were cut with straight microscissors. (e1) LPC in saline was injected into the sciatic nerve via a 30 gauge Hamilton syringe. (e2) The nerve tissue is slightly expanded locally after injection, with a pinhole visible in the epineurium.

Rats were placed under general anesthesia by intraperitoneal injection of sodium pentobarbital (50 mg/kg). Approximately 0.5 cm below the midpoint of the femur in rats, the skin was cut parallel to the femur and the biceps femoris muscle was bluntly separated to expose the sciatic nerve trunk. In the control group, the sciatic nerve was kept nonoperated (Figure 1a). In the transverse group, the sciatic nerve trunk was cut with straight microscissors (Figure 1(b1)). To preserve the original anatomical structure, the severed ends of both nerves were placed close together and the epineurium was fixed with 9/0 nonabsorbable sutures (Figure 1(b2)). In the clamp group, a crush lesion was inflicted at the sciatic nerve trunk using microforceps and pressure was maintained for 30 s. This procedure resulted in the section of all axons but spared the epineurium, leaving a 1 mm wide gap (Figure 1c). In the keep epineurium group, the sciatic nerve epineurium was slit longitudinally and axons were cut with straight microscissors (Figure 1(d1 and d2)). In the chemical damage group, 5 µL of 2% LPC (Sigma-Aldrich) in saline was injected into the sciatic nerve via a 30 gauge Hamilton syringe, as previously described [16,17] (Figure 1(e1 and e2)). The surgical wound was closed with 4-0 nylon sutures, and the rats were returned to their cages. The whole process was carried out under aseptic conditions. The recovery of the rats was monitored for one month following damage model construction. They were then evaluated using behavioral analyses and electrophysiological and histological assessment methods.

### 2.3 Evaluations

#### 2.3.1 Electrophysiological measurements

Before the operation, immediate postoperative, two and four weeks postoperatively, electrophysiological measurements of all rats that had not been sacrificed at each time point were performed. Under anesthesia with compound anesthetic, the previous surgical site at the right mid-thigh level was reopened and the sciatic nerve was reexposed. Electrical stimuli (1.2 mA in strength) were applied to the sciatic nerve trunk at the proximal end. Compound muscle action potentials (CMAPs) were recorded on the gastrocnemius belly. We recorded the effects of three repetitive measures per individual rat. CMAPs were expressed as the mean ± SD per group.

#### 2.3.2 Walking footprint analysis

Before the operation, one, two, three, and four weeks postoperation, walking track assessments of all animals that had not been sacrificed at each time point were performed. Briefly, the walking footprint was analyzed using a DigiGait System (Mouse Specifics, Framingham, MA, USA). Rats were trained at a speed of 25 cm/s before recording and then tested at a speed of 25 cm/s. For each test, at least 2 s of continuous walking were recorded. Footprints were captured and analyzed using DigiGait analysis software (DigiGait 12.4). The sciatic functional index (SFI) for each time point was calculated using previous formulas: SFI = −51.2 × [(EPL − NPL)/NPL] + 118.9 × [(ETS − NTS)/NTS] − 7.5, where EPL is the experimental print length, NPL is the normal print length, ETS is experimental toe spread, and NTS is normal toe spread [18].

#### 2.3.3 Hot-plate test

Before the operation, one, two, three, and four weeks postoperation, the hot-plate test was used to measure thermal withdrawal latency as an index of the nociceptive threshold at each time point [19]. Briefly, the right hind paws of the rats were placed on a hot-plate (Constant Temperature Heating Plate; Shanghai Kuncheng Scientific Instrument Ltd, China) heated to 50°C. The latency time was defined as the period between the animal’s initial placement on the hot-plate surface and the time when the animal licked its paws, jumped to avoid a thermal nociceptive stimulus, and/or urinated. To minimize tissue damage, a cutoff time of 60 s was adopted. We recorded the effects of three repetitive measures per individual rat performed at a minimum of 2 min apart. The thermal withdrawal time (seconds) was expressed as the mean ± SD per group.

#### 2.3.4 Calibrated forceps test

Before the operation, one, two, three, and four weeks postoperation, the calibrated forceps test was used to evaluate the mechanical threshold response at each time point. This algometer allows calibrated forceps (Rodent pincher-analgesia meter; Bioseb *In Vivo* Research Instruments, Vitrolles, France) to induce quantifiable mechanical stimulation in the rat. As previously described by Luis-Delgado et al. [20], we recorded the effects of three repetitive measurements on each right hind paw to provide a sensitive and reliable way of testing the mechanical threshold. The force applied to the foot was increased incrementally and slowly until the paw was withdrawn. The test interval was 5 min. To minimize tissue damage, a cutoff force of 10 newtons was adopted. The maximum force applied to the paw at the time of withdrawal was recorded as displayed by a dynamometer in newton (N). Withdrawal latency was expressed as the mean ± SD per group.

#### 2.3.5 Gastrocnemius muscle wet weight and Masson’s trichrome staining

Two and four weeks after surgery, three rats were randomly selected and killed by cervical dislocation in each group after electrophysiological evaluation. The double lower limbs were cut open and the intact gastrocnemius was cut off. The bilateral gastrocnemius muscles were blotted with absorbent paper, evaluated for wet muscle weight (WMW), and recorded. The remaining rate of WMW was defined as follows: WMW in the operation side/WMW in the healthy side × 100%.

Next, muscles were fixed in 4% paraformaldehyde, dehydrated with a serial gradient of ethanol, and cleared in xylene. Afterward, specimens were embedded in paraffin and sectioned into 5 µm slices, which were dyed with Masson’s trichrome (Solibor, Beijing, China) and observed under a microscope (Leica). Digital images were captured, and muscle fiber areas were calculated using Image-Pro Plus 6.0 software. Fifty muscle fibers were counted in each sample, and the area was expressed as the mean ± SD per group.

#### 2.3.6 Immunofluorescence

At two and four weeks postsurgery, a 5 mm segment from the nerve was harvested from the distal end of the operation site. After fixation with 4% paraformaldehyde for 24 h and dehydration with sucrose solution (20%) at 4°C for 24–48 h, nerve samples were embedded with the optimal cutting temperature compound and sliced into 8 µm thick frozen transverse sections. Sections were then rinsed with phosphate-buffered saline and 0.3% Triton X-100 and blocked with 0.5% bovine serum albumin sealing fluid. Nerve sections were subsequently incubated with rabbit anti-neurofilament heavy (NF-H) polypeptide (1:500; Abcam) or rabbit anti-S100β (1:200; Abcam) antibody overnight at 4°C. After washing, the sections were incubated with Alexa Fluor 488- or Alexa Fluor 594-conjugated goat anti-rabbit IgG (Abcam) secondary antibodies. Cell nuclei were counterstained with 4′,6-diamidino-2-phenylindole (DAPI). Slides were viewed and imaged using fluorescence microscopy (Leica).

#### 2.3.7 Electron microscopy

At two and four weeks postsurgery, the nerve tissues from the distal end of the operation site were fixed in 4% glutaraldehyde for 3 h. The specimens were postfixed with osmium tetroxide and immersed in Epon812 epoxy resin (Fluka, Buchs, Germany). Segmented nerves were subsequently cut into 1 µm semithin sections and mounted on slides, which were dyed with 1% toluidine blue and imaged (Reichert Ultracut S Wild M3z, Leica). Toluidine blue-stained images were used to count the number of myelinated nerve fibers. Myelin was quantified using Image-Pro Plus 6.0 software.

### 2.4 Statistical analysis

All data are presented as the mean ± standard error of the mean (SEM). GraphPad Prism 6.0 (USA) was used for statistical analysis and draft graphs. The data were analyzed by two-way analysis of variance (ANOVA) with factors of treatment and time. Intergroup differences were analyzed by performing *post hoc* Bonferroni tests. *P* values ≤0.05 were considered statistically significant (### or ***, *P* ≤ 0.001; **, *P* ≤ 0.01; and *, *P* ≤ 0.05).

## 3 Results

### 3.1 Electrophysiological measurements

To measure electrical conduction in different ways of damaging the SNI model, MAPs were recorded from the right gastrocnemius muscle in response to electrostimulation. The amplitude recorded potentials were measured to evaluate the effectiveness of nerve conduction.

Regarding the different damage ways of the SNI model, two-way ANOVA showed significant interactions between different damage ways and times after SNI for MAP amplitude (*F* (12,45) = 8.538; *p* < 0.0001). Sciatic nerve stimulation produced weak MAPs in the clamp group, the keep epineurium group, and the chemical damage group immediately after injury, and no signal was recorded in the transverse group. *Post hoc* Bonferroni tests indicated significant differences when compared with the control group for amplitude (24.18 ± 2.59 mV; *p* < 0.0001). There were no significant differences in amplitude between the different groups (*p* > 0.05).

After two weeks, the amplitude of the chemical damage group reached 11.55 ± 2.02 mV, and *post hoc* Bonferroni tests showed significant differences in amplitude when the chemical damage group was compared with the transverse group (0.80 ± 0.40 mV, *p* < 0.001) and the clamp group (3.53 ± 1.67 mV, *p* < 0.05). *Post hoc* Bonferroni tests indicated significant differences when injury groups were compared with the control group for amplitude (21.18 ± 2.32 mV; *p* < 0.01).

**Figure 2:**
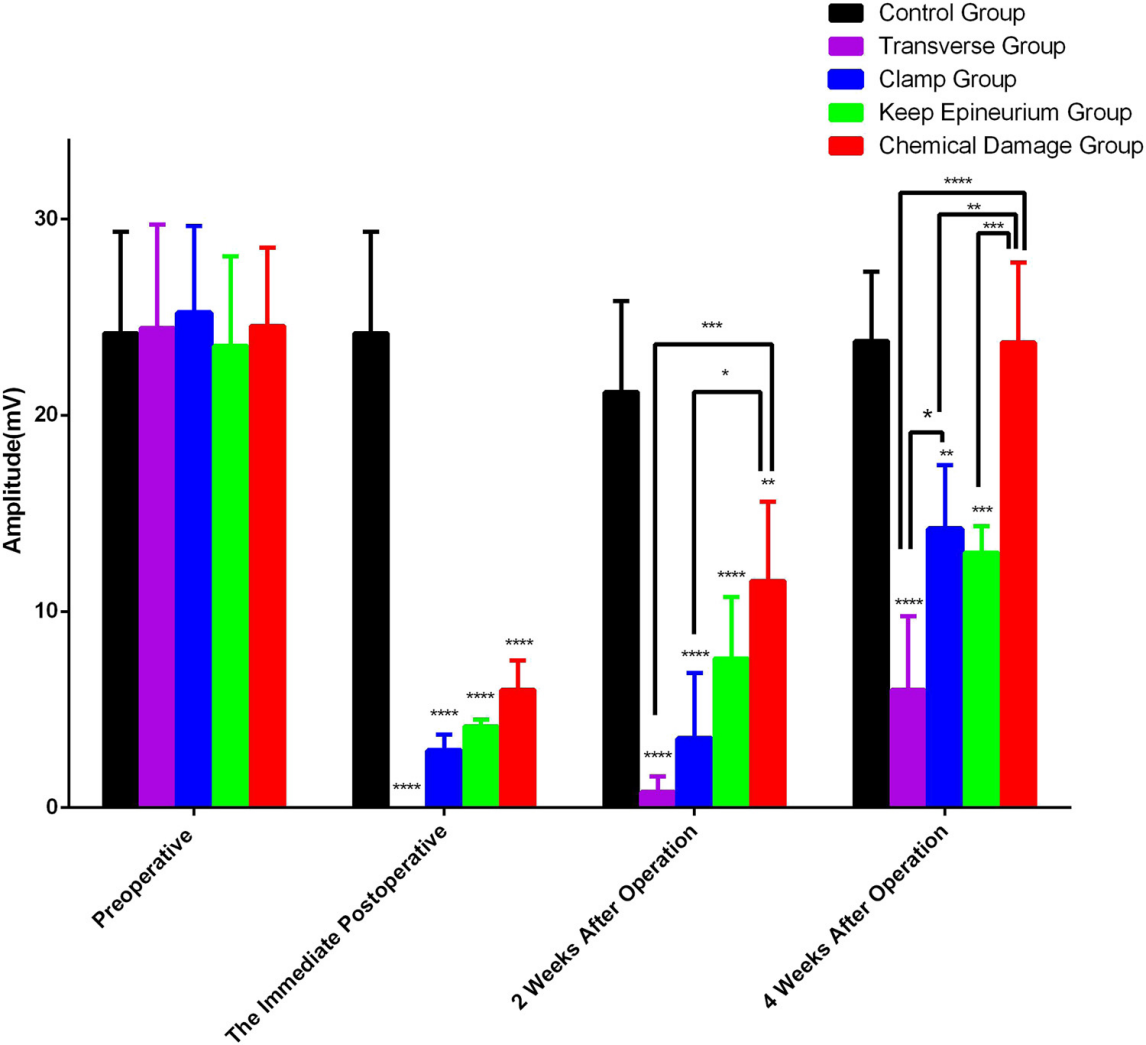
MAP amplitude at the immediate postoperative, two, and four weeks after injury model construction. The time effect (*F* (3,45) = 105.9; *p* < 0.0001), the treatment effect (*F* (4,15) = 33.20; *p* < 0.0001), and interaction between time and treatment (*F* (12,45) = 8.538; *p* < 0.0001) were significant. ****P < 0.0001, ***P < 0.001, **P < 0.01, and *P < 0.05.

At the end of four weeks after injury, *post hoc* Bonferroni tests showed significant differences in amplitude when the transverse group (6.00 ± 1.87 mV, *p* < 0.0001), the clamp group (14.20 ± 1.63 mV, *p* < 0.01), and the keep epineurium group (13.00 ± 0.67 mV, *p* < 0.001) were compared with the control group (23.78 ± 1.76 mV). There were no significant differences in amplitude between the control group and the chemical damage group (23.70 ± 2.04 mV, *p* > 0.05). *Post hoc* Bonferroni tests indicated significant differences in amplitude when the chemical damage group was compared with the transverse group (*p* < 0.0001), the clamp group (*p* < 0.01), and the keep epineurium group (*p* < 0.001) (Figure 2).

### 3.2 Walking footprint analysis

**Figure 3:**
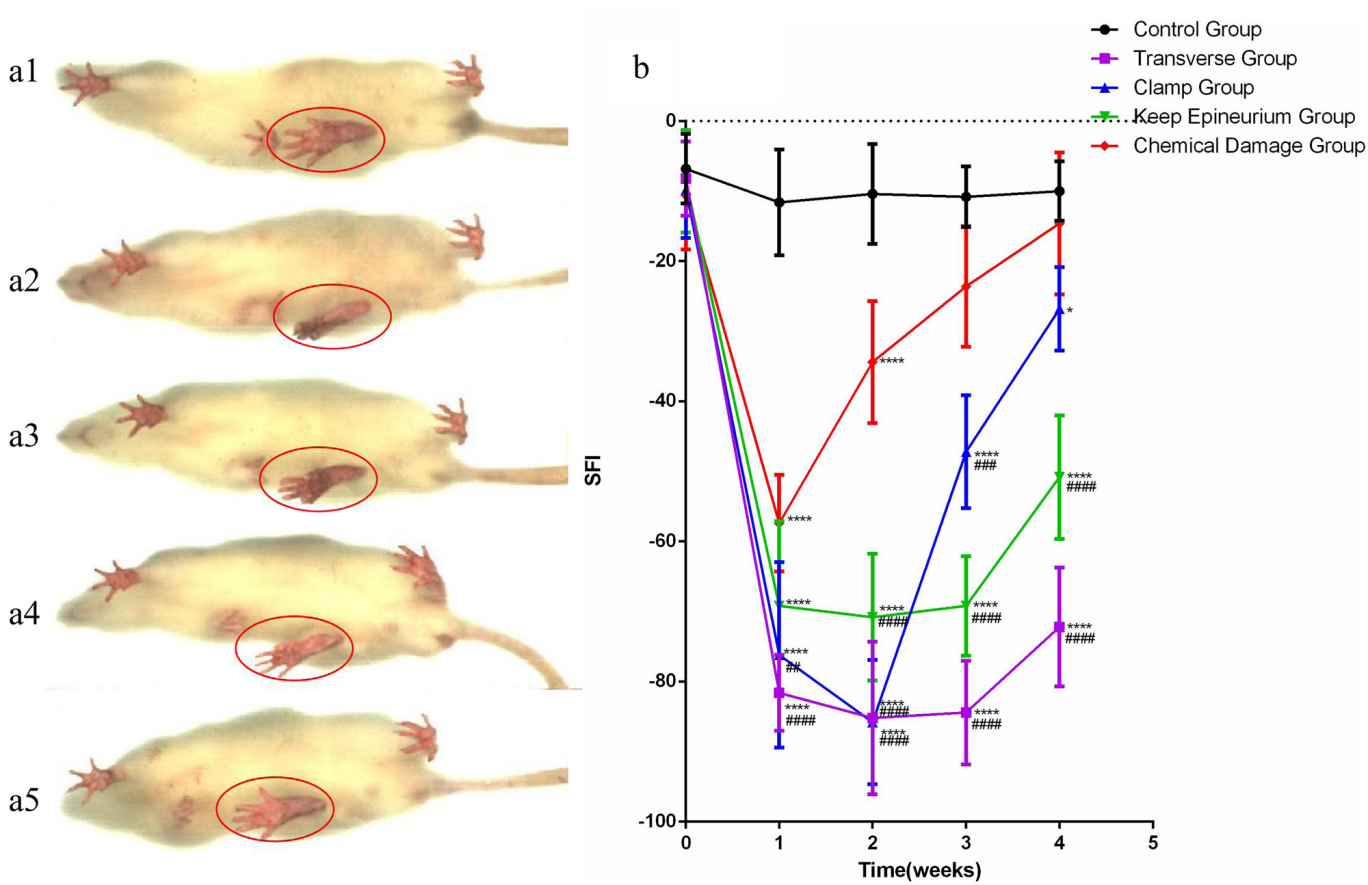
The paw area and the sciatic nerve functional index curve. (a1) Representative gait image and paw area of the control group four weeks after surgery. (a2) Transverse group. (a3) Clamp group. (a4) Keep epineurium group. (a5) Chemical damage group. (b) Analysis of the recovery of motor function by the sciatic nerve functional index curve. *P < 0.05, **P < 0.01, ***P < 0.001, ****P < 0.0001 vs the control group; ##*P* < 0.01, ###*P *< 0.001, ####*P* < 0.0001 vs the chemical damage group. Data are expressed as the mean ± SD (number of footprints from each group = 5; two-way ANOVA analysis followed *post hoc* multiple comparisons with a Bonferroni correction). SFI: sciatic function index.

To compare the functional recoveries, we performed a walking track test. The SFI was detected through DigiGait every week. Regarding the different damage methods of the SNI model, two-way ANOVA showed significant interactions between different damage methods and times after SNI for SFI (*F* (16,80) = 18.59; *p* < 0.0001). After one week, the four injury groups showed a decrease in the movement function; the most significant decline in the movement function was observed in the transverse group, and the least significant decline in the movement function was observed in the chemical damage group. For one and four weeks, the SFI of the transverse group was always in a low state and increased slightly at the end; the SFI of the clamp group reached the lowest value at two weeks, then rose rapidly and reached a high value only second to the chemical damage group; the SFI of the keep epineurium group kept a low value from one week to three weeks and increased at four weeks; and the SFI of the chemical damage group rose rapidly and reached the control group level (Figure 3).

### 3.3 Hot-plate test

**Figure 4:**
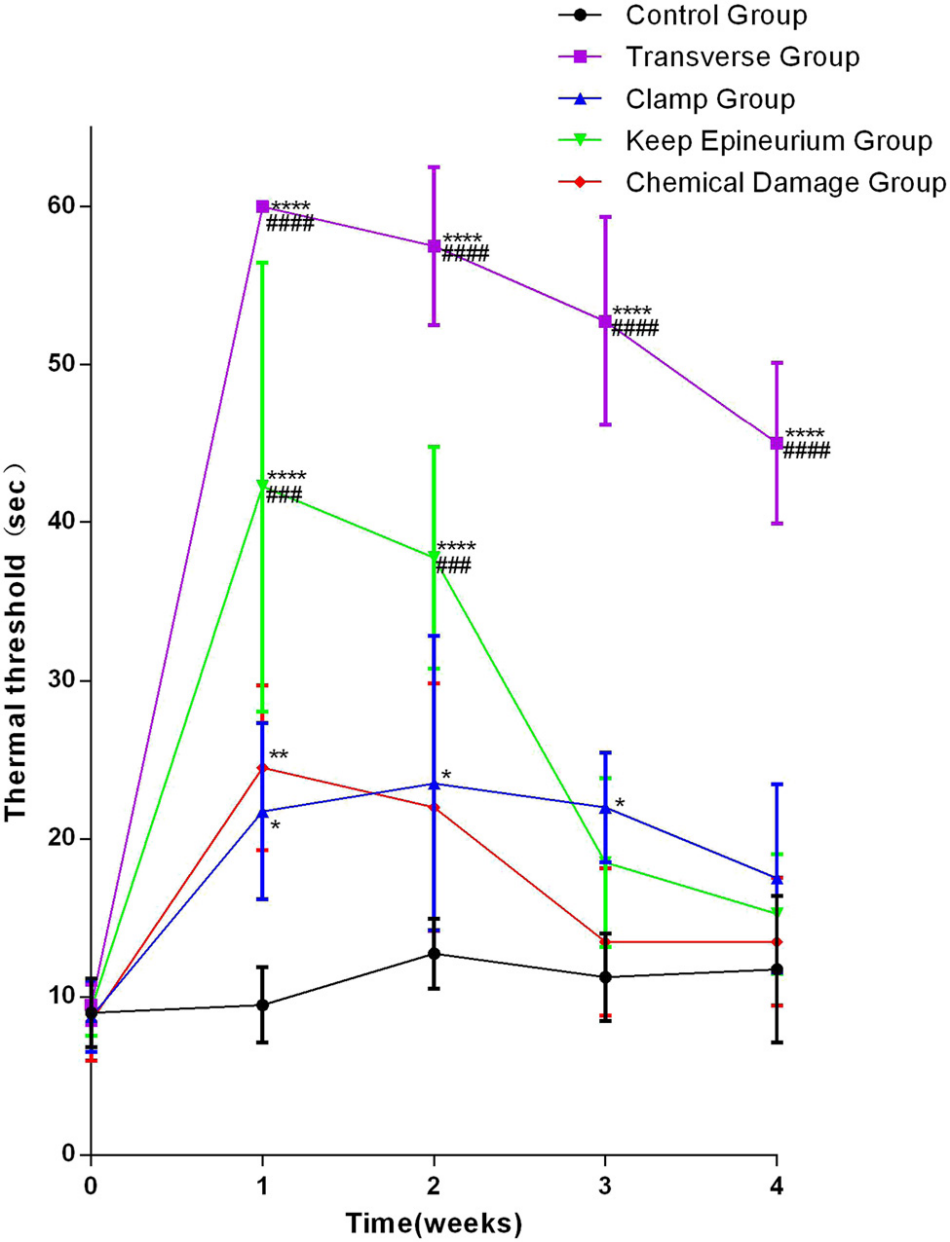
Analysis of the thermal threshold by thermal withdrawal time curve. *P < 0.05, **P < 0.01, ***P < 0.001, ****P < 0.0001 vs the control group; ##*P* < 0.01, ###*P* < 0.001, ####*P* < 0.0001 vs the chemical damage group. Data are expressed as the mean ± SD (number from each group = 5; two-way ANOVA analysis followed *post hoc* multiple comparisons with Bonferroni correction).

To examine the influence of different SNI models on nociceptive pathways, thermal tests were performed at each time point. Regarding the different damage methods of the SNI model, two-way ANOVA showed significant interactions between different damage methods and times after SNI for thermal withdrawal time (*F* (16,60) = 10.22; *p* < 0.0001). After one week, the four injury groups showed prolonged thermal withdrawal time, and the most significantly prolonged thermal withdrawal time was observed in the transverse group. For one and four weeks, the thermal withdrawal time of the transverse group gradually decreased but was still the longest at the end; the thermal withdrawal time of the clamp group was least prolonged and recovered slowly; the thermal withdrawal time of the keep epineurium group recovered quickly; and the thermal withdrawal time of the chemical damage group was less prolonged, recovered quickly, and reached the control group level (Figure 4).

### 3.4 Calibrated forceps test

**Figure 5:**
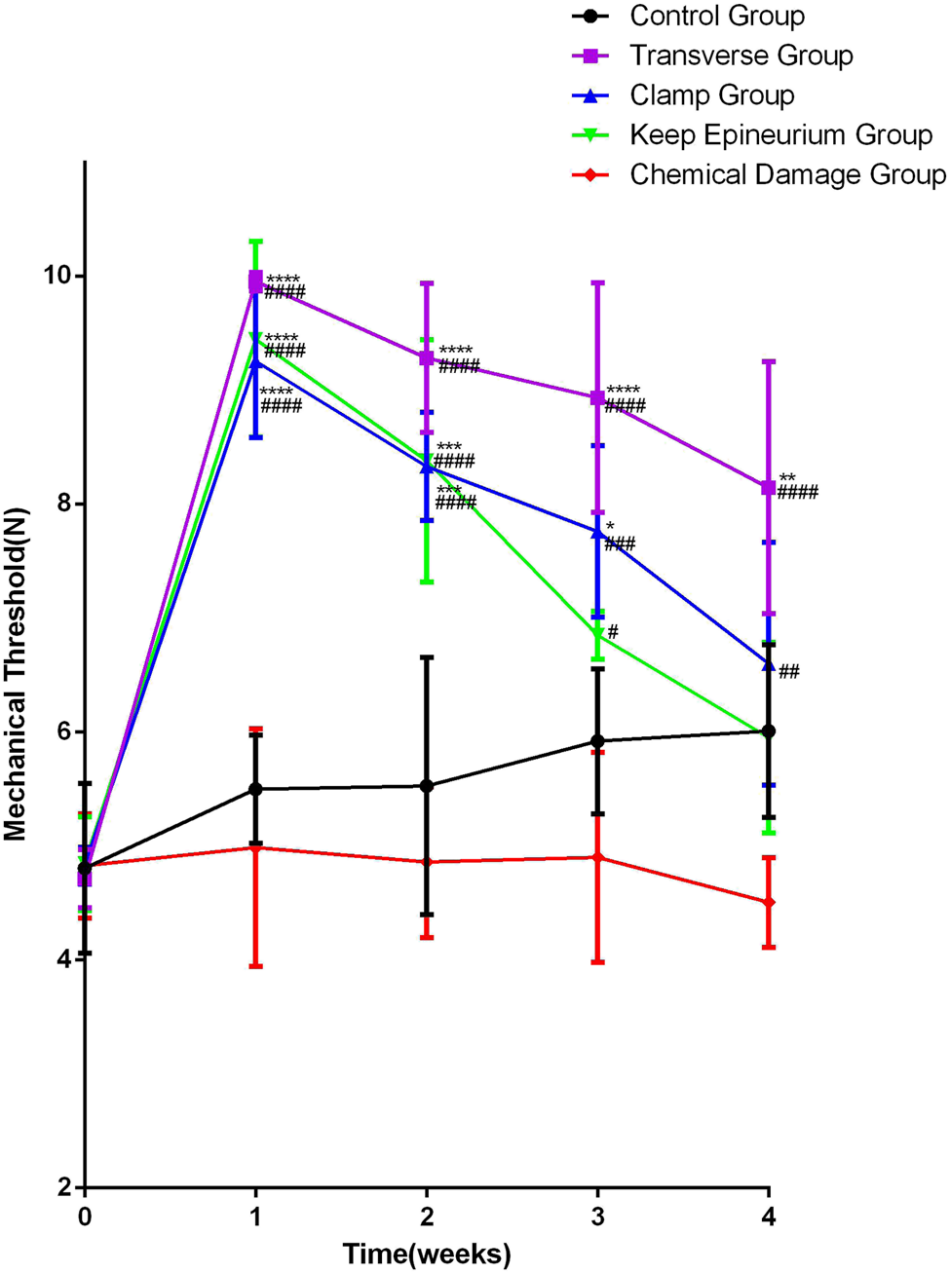
Analysis of the mechanical threshold by the withdrawal threshold curve. *P < 0.05, **P < 0.01, ***P < 0.001, ****P < 0.0001 vs the control group; ##*P* < 0.01, ###*P* < 0.001, ####*P* < 0.0001 vs the chemical damage group. Data are expressed as the mean ± SD (number from each group = 3; two-way ANOVA analysis followed *post hoc* multiple comparisons with Bonferroni correction).

To examine the influence of different SNI models on nociceptive pathways, calibrated forceps tests were performed at each time point. Regarding the different damage methods of the SNI model, two-way ANOVA showed significant interactions between different damage methods and times after SNI for the mechanical threshold (F (16,40) = 5.516; *p* < 0.0001). After one week, the injury group, except the chemical damage group, showed an increase in the mechanical threshold, and the most significant increase in the mechanical withdrawal threshold was observed in the transverse group. For the one and four weeks, the mechanical withdrawal threshold of the transverse group gradually decreased but was still the highest at the end; the mechanical withdrawal threshold of the clamp group increased significantly and recovered quickly; the mechanical withdrawal threshold tendency of the keep epineurium group was similar to that of the clamp group; and the mechanical withdrawal threshold level of the chemical damage group was inferior to that of the control group. However, there were no significant differences in the mechanical withdrawal threshold between the chemical damage group and the control group (Figure 5).

### 3.5 Gastrocnemius muscle wet weight and Masson’s trichrome staining

**Figure 6:**
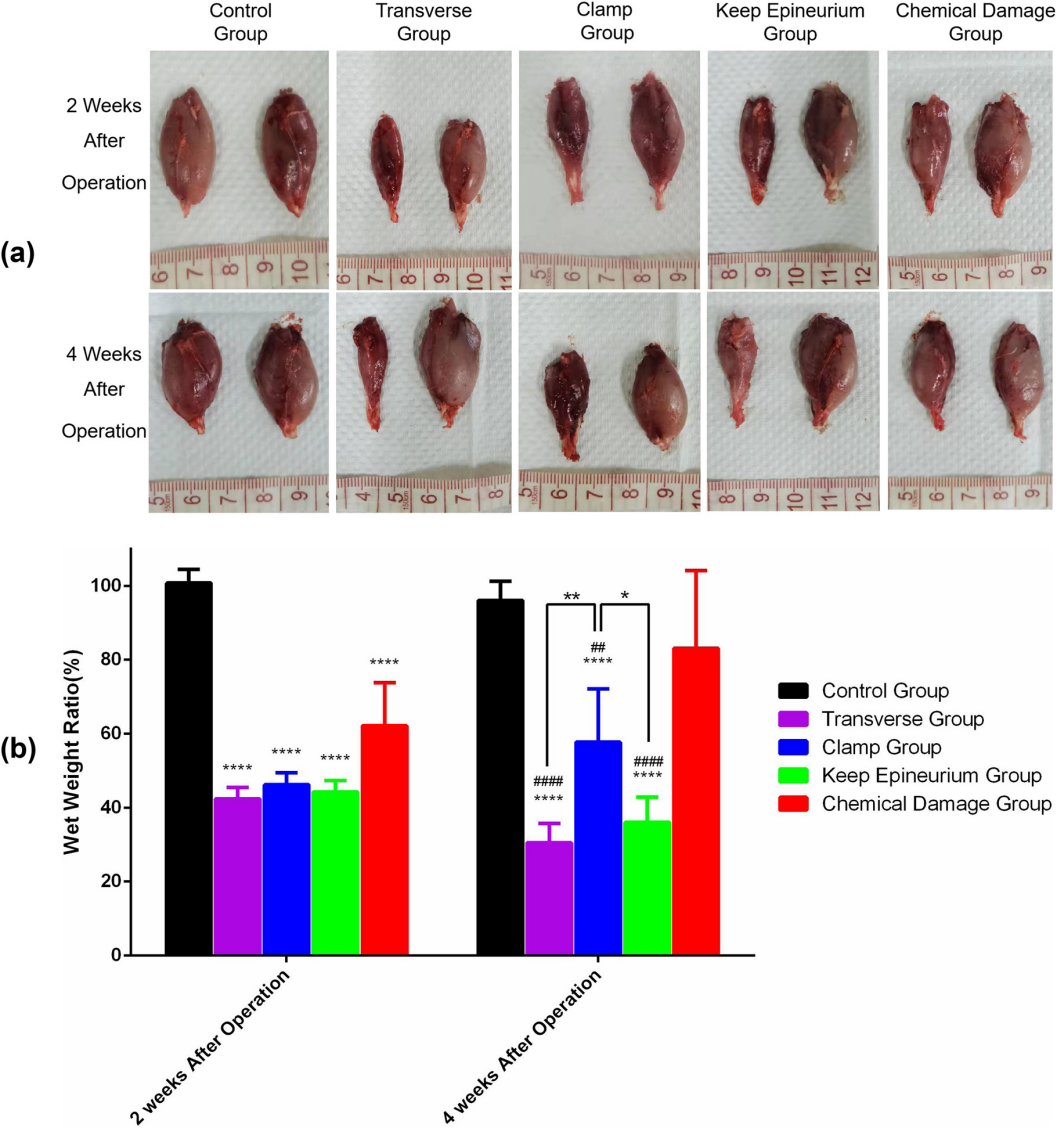
General observation of the gastrocnemius muscle at two and four weeks postsurgery and assessment of the wet weight ratio of the gastrocnemius muscle at two and four weeks postsurgery. (a) General observation at two and four weeks after surgery. (b) Wet weight ratios were measured to evaluate functional recovery of the gastrocnemius muscle. *P < 0.05, **P < 0.01, ***P < 0.001, ****P < 0.0001 vs the control group; ##*P* < 0.01, ####*P* < 0.0001 vs the chemical damage group. Data are expressed as the mean ± SD (*n* = 4; two-way ANOVA analysis followed *post hoc* multiple comparisons with Bonferroni correction).

**Figure 7:**
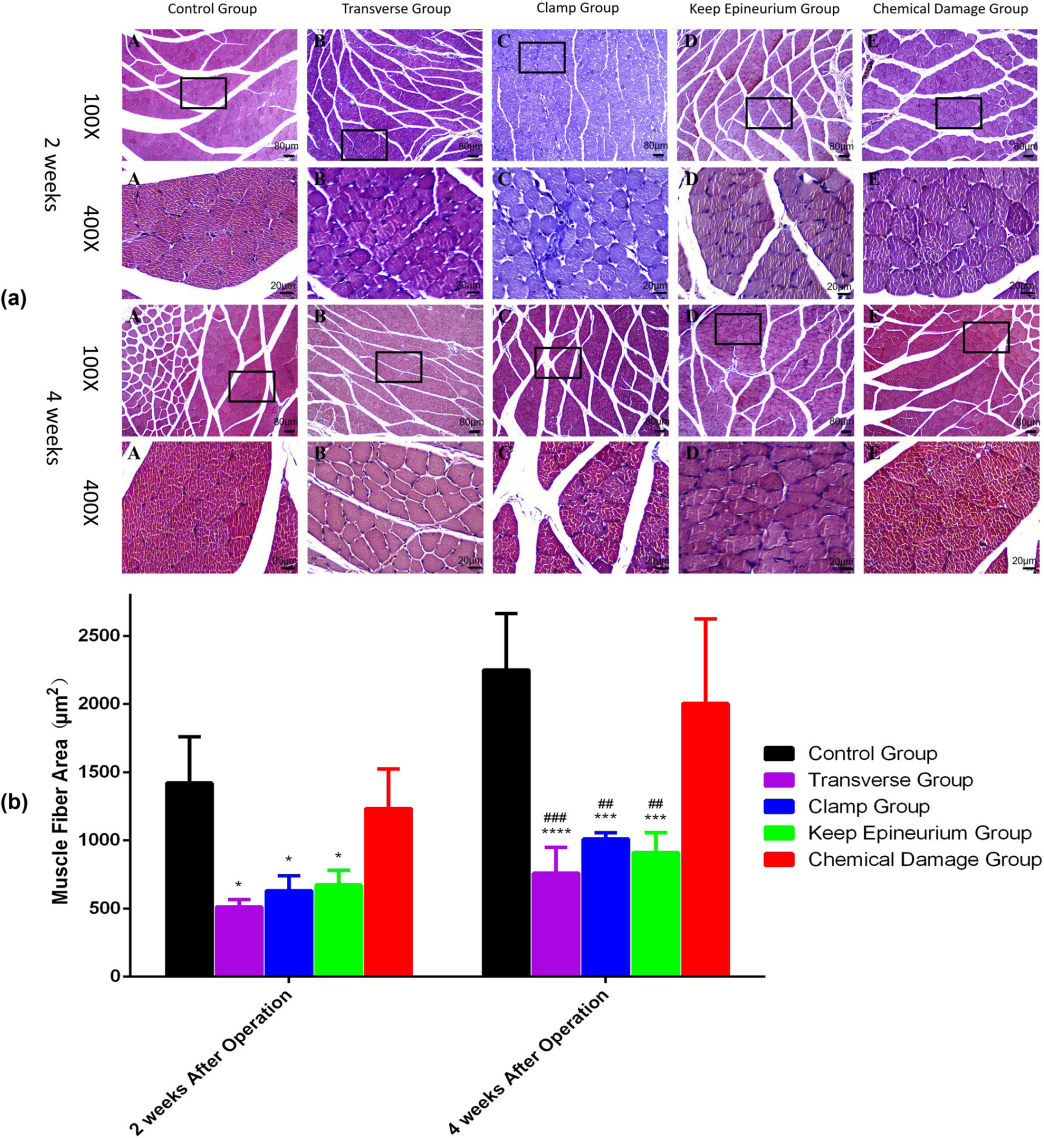
Masson’s trichrome staining of the gastrocnemius muscle from the experimental side at two and four weeks postsurgery and assessment of the muscle fiber area at two and four weeks postsurgery. (a) Cross-sections with Masson’s trichrome staining at two and four weeks after surgery. 100×, scale bar: 100 µm. 400×, scale bar: 25 µm. (b) Gastrocnemius muscle fiber areas were measured to evaluate the functional recovery of the gastrocnemius muscle. *P < 0.05, ***P < 0.001, ****P < 0.0001 vs the control group; ##*P* < 0.01, ###*P* < 0.001 vs the chemical damage group. Data are expressed as the mean ± SD (*n* = 3; two-way ANOVA analysis followed *post hoc* multiple comparisons with Bonferroni correction).

Recovery of the gastrocnemius muscle from atrophy was assessed by the wet weight of the gastrocnemius muscle ratio. Recovery was also evaluated by calculating the muscle fiber area of sections with Masson’s trichrome staining. According to the macroscopic appearance of gastrocnemius muscles (Figure 6a) and Masson’s trichrome-stained sections (Figure 7a) at two and four weeks postsurgery, different extents of atrophy existed in the four injury model groups. Two weeks postsurgery, gastrocnemius muscle wet weight ratios were notably decreased in the four injury model groups (*P* < 0.0001). However, there were no significant differences among the four injury model groups. Four weeks postsurgery, *post hoc* Bonferroni tests showed significant differences in wet weight ratios when the transverse group (30.46 ± 2.61, *p* < 0.0001), the clamp group (57.72 ± 7.22, *p* < 0.01), and the keep epineurium group (36.01 ± 3.38, *p* < 0.0001) were compared with the control group (96.08 ± 2.58) and the chemical damage group (83.15 ± 10.51) (Figure 6b). Sections with Masson’s trichrome staining showed that the gastrocnemius muscle fiber area was reduced in the injury model groups. Areas of muscle fiber were smaller in the transverse group (511.47 ± 31.47 µm^2^, *p* < 0.05), the clamp group (631.00 ± 63.06 µm^2^, *p* < 0.05), and the keep epineurium group (671.27 ± 61.91 µm^2^, *p* < 0.05) compared with the control group (1420.57 ± 195.76 µm^2^) and the chemical damage group (1233.47 ± 167.21 µm^2^) at two and four weeks postsurgery (Figure 6b).

### 3.6 Immunofluorescence

**Figure 8:**
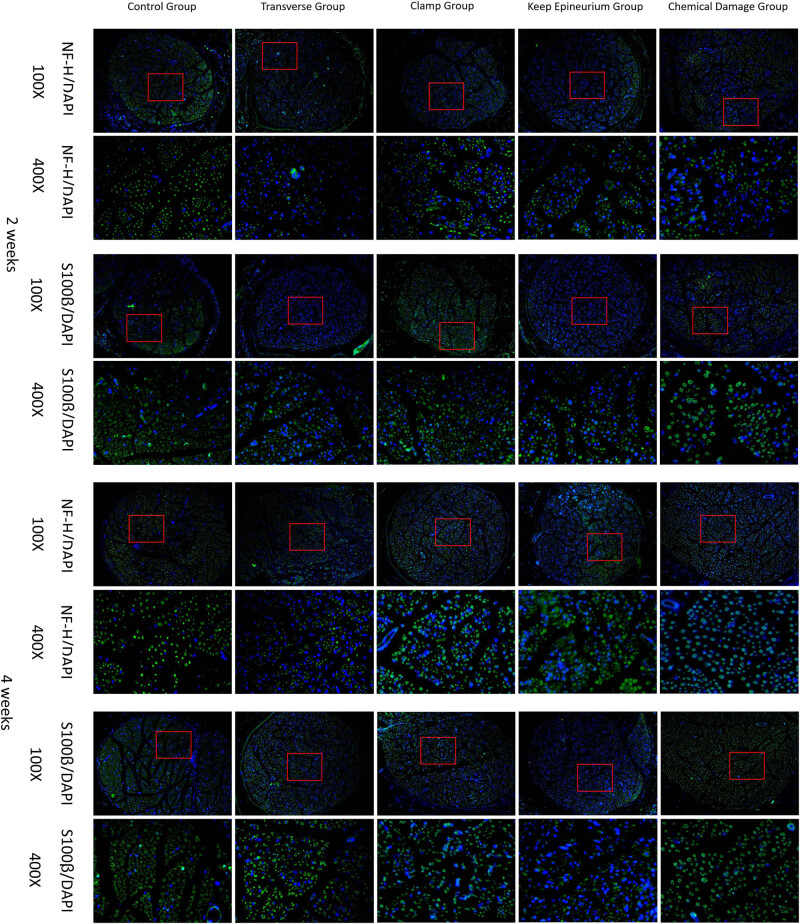
Immunofluorescence staining and distribution of nerve lesions at two and four weeks postsurgery. NF-H (green); S100β (green); and DAPI (blue). 100× (scale bar: 100 µm), 400× (scale bar: 25 µm).

Figure 8 shows NF-H and S100β immunofluorescence staining of nerves at two and four weeks postsurgery. In the control group, NF-H-expressing axons and S100β-expressing myelin sheaths were evenly distributed at two and four weeks postsurgery. In the transverse group and the keep epineurium group, the distributions of NF-H-expressing axons and S100β-expressing myelin sheaths were not uniform and had a small scope at two weeks postsurgery but were improved at four weeks postsurgery. In the chemical damage group, the distributions of NF-H-positive axons and S100β-positive myelin sheaths were not uniform but had a larger scope at two weeks postsurgery but were improved at four weeks postsurgery. In the clamp group, the distributions of NF-H-expressing axons and S100β-expressing myelin sheaths were uniform but with a larger scope at two weeks. However, NF-H-expressing axons and S100β-expressing myelin sheaths were uniformly distributed in the clamp group at four weeks postsurgery.

### 3.7 Electron microscopy

**Figure 9:**
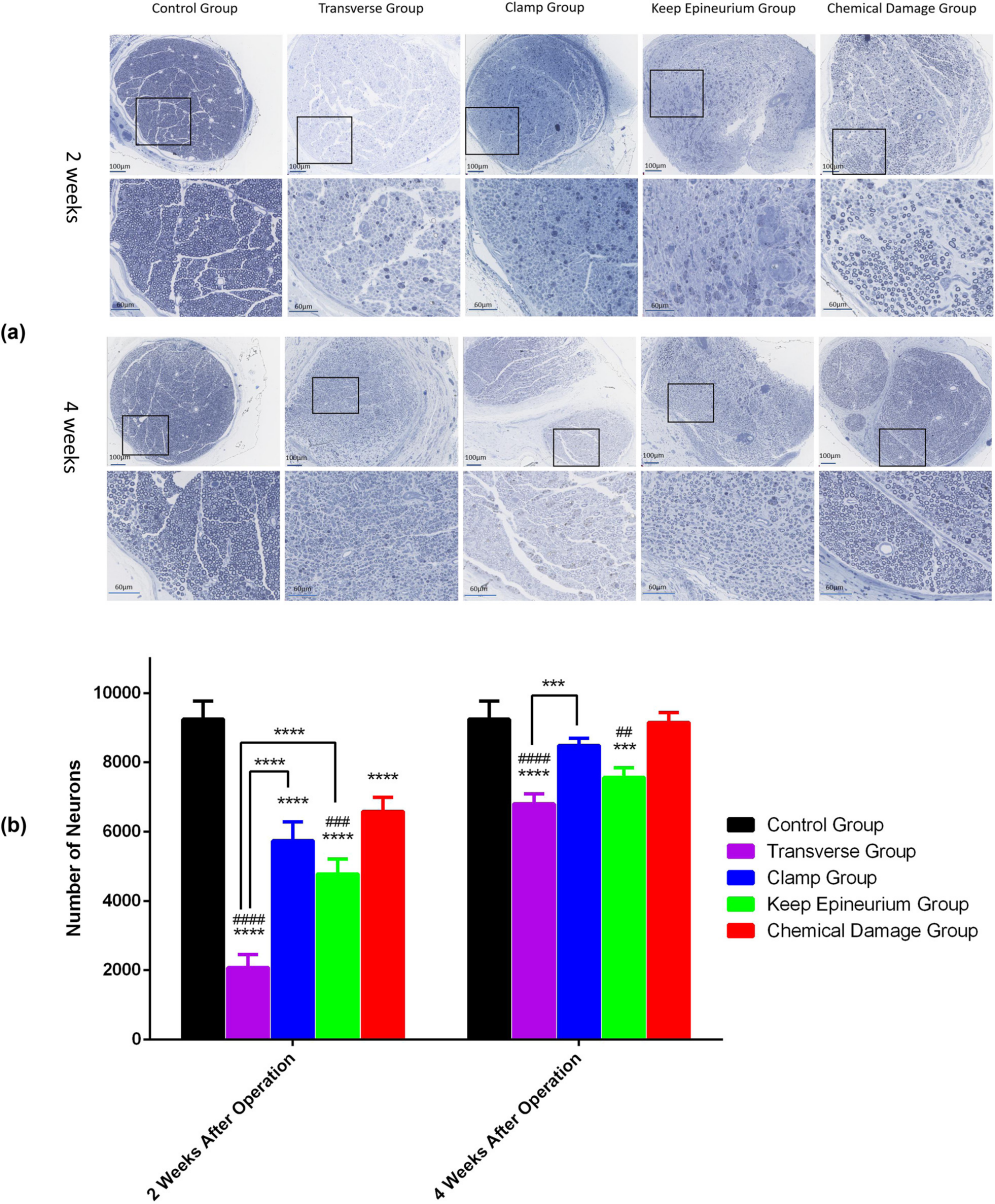
Toluidine blue staining and myelinated nerve fiber numbers of sections at two and four weeks postsurgery. (a) Toluidine blue staining at two and four weeks postsurgery. (b) Myelinated nerve fiber numbers were measured to evaluate nerve recovery. ****P < 0.0001, ***P < 0.001 vs the control group; ##*P* < 0.01, ###*P* < 0.001, ####*P* < 0.0001 vs the chemical damage group. Data are expressed as the mean ± SD (*n* = 3; two-way ANOVA analysis followed *post hoc* multiple comparisons with Bonferroni correction).

To further investigate nerve recovery, the numbers of myelinated nerve fibers in the nerve segment groups were counted (Figure 9a). At two and four weeks postsurgery, myelinated nerve fibers from the control group were uniformly distributed. At two weeks postsurgery, the numbers of myelinated nerve fibers were notably decreased in the four injury model groups (*P* < 0.0001). In the transverse group, the numbers of myelinated nerve fibers were significantly different from those in the other injury model groups at two weeks after surgery. *Post hoc* Bonferroni tests indicated significant differences when the keep epineurium group was compared with the chemical damage group for the number of myelinated nerve fibers (*P* < 0.001). At four weeks postsurgery, the numbers of myelinated nerve fibers were notably decreased in the transverse group and the keep epineurium group (*P* < 0.01). There were no significant differences in the numbers of myelinated nerve fibers among the control group, the clamp group, and the chemical damage group (*p* > 0.05) (Figure 9).

## 4 Discussion

SNI is one of the most common peripheral nerve injuries. After this nerve injury, partial loss of the function of the innervating region may occur, resulting in motor and sensory dysfunction. When the injury is serious, neurotrophic changes may occur, resulting in paralysis of the innervated muscles and loss of joint function. Neural structures are supported by three layers of connective tissue: endoneurium, perineurium, and epineurium. In our study, we constructed a new model of SNI that kept the epineurium and other nerve tissue inside the epineurium was cut. We want to know the importance of the three layers of connective tissue by comparing this model with the clamp model. In the sciatic nerve clamp injury model, the continuity of all axons can be interrupted without interrupting the connective tissue of the nerve (especially the epineurium), and the continuity of the nerve trunk is retained [21,22]. The proximal and distal ganglion segments of the lesion remain connected, allowing the severed axon to regenerate along the optimal regeneration path, achieving the goal of primitive innervation [6]. The difference between clamp injury and keep epineurium injury is whether the endoneurium and perineurium are retained or not. In our results, there were no significant differences in the numbers of myelinated nerve fibers, muscle fiber area, wet weight ratio of gastrocnemius muscle, and muscle action potentials between clamp injury and keep epineurium injury. From these results, we can speculate that grouped fascicular suture is of little clinical significance. Some studies have the same point [23,24], but our study can eliminate the problem of suture bundle dislocation. With advances in microscopy, we have been able to reconstruct nerves at the fascicular level [25], but this method is complex and meaningless.

Chemical injury is a method of causing peripheral nerve injury by injecting chemical drugs into or around the sciatic nerve. The chemical drugs used include antibiotics [26] and phospholipids [17]. Antibiotics mimic neuropathy, a condition of inflammation or infection, by injecting them around nerves and causing perineuroinflammation. Phosphoesters are usually synthesized after peripheral nerve injury and can be converted to lysophosphatidic acid, which subsequently leads to demyelination and neuropathic pain (ectopic pain or hyperalgesia) through an unknown process [27,28]. From the results of our study, chemical injury leads to demyelination and hyperalgesia, but some studies indicated that chemical models showed plantar hypoalgesia [16,29]. The cause for this discrepancy requires further investigation. In our study, chemical injury led to incomplete demyelination, and the toluidine blue staining of the chemical damage group showed many mature myelin sheaths at two weeks postsurgery. In conclusion, chemical injury models self-recover quickly and damage incompletely.

Transverse injury corresponds to the Sunderland nerve injury classification V, which is the most severe type of nerve injury. Some scholars believe that the transverse injury of nerves not only causes damage to motor functions but also leads to autonomic nerve dysfunction [30]. Our results show that it has the most stable effect among all damage models.

Our study has several limitations. First, due to the time and space constraints of our study, the sample size was small and bias may have occurred. Second, we only observed four weeks and the long-term prognosis is unclear. Third, we only carried out animal experiments and did not further explore the mechanism. Future studies are needed to address these limitations.

In conclusion, transverse injury models have the most stable effect among all damage models; chemical injury models self-recover quickly and damage incompletely with poor stability of effect; and clamp injury models and keep epineurium injury models have no significant differences in many ways with medium stability.
