# Erratum to “Construction and effect evaluation of different sciatic nerve injury models in rats”

**DOI:** 10.1515/tnsci-2022-0246

**Published:** 2022-09-06

**Authors:** Siwei Qu, Ning Ma, Weixin Wang, Sen Chen, Qi Wu, Yangqun Li, Zhe Yang

**Affiliations:** 2nd Department, Plastic Surgery Hospital, Chinese Academy of Medical Sciences and Peking Union Medical College, No. 33 Badachu Road, Shijingshan District, Beijing 100144, China

In the published article “Construction and effect evaluation of different sciatic nerve injury models in rats” (https://doi.org/10.1515/tnsci-2022-0214), the first author’s name and family name are given in reverse. The authors would like to correct this error and apologize for any inconvenience it may have caused. The correct format of the authors’ list is as follows:

Siwei Qu, Ning Ma, Weixin Wang, Sen Chen, Qi Wu, Yangqun Li, Zhe Yang

